# Acupuncture for pain in endometriosis

**DOI:** 10.1590/1516-3180.20131316T1

**Published:** 2013-12-01

**Authors:** Xiaoshu Zhu, Kindreth D. Hamilton, Ewan D. McNicol

## Abstract

**BACKGROUND:**

Endometriosis is a prevalent gynaecological condition, significantly affecting women's lives. Clinical presentations may vary from absence of symptoms to complaints of chronic pelvic pain, most notably dysmenorrhoea. The management of pain in endometriosis is currently inadequate. Acupuncture has been studied in gynaecological disorders but its effectiveness for pain in endometriosis is uncertain.

**OBJECTIVE:**

To determine the effectiveness and safety of acupuncture for pain in endometriosis.

**METHODS:**

Search methods: We searched the Cochrane Menstrual Disorders and Subfertility Group (MSDG) Specialized Register of controlled trials, Cochrane Central Register of Controlled Trials (CENTRAL) (The Cochrane Library), MEDLINE, EMBASE, CINAHL, AMED, PsycINFO, CNKI and TCMDS (from inception to 2010) and reference lists of retrieved articles. *Selection criteria:* Randomized single or double-blind controlled trials enrolling women of reproductive age with a laparoscopically confirmed diagnosis of endometriosis and comparing acupuncture (body, scalp or auricular) to either placebo or sham, no treatment, conventional therapies or Chinese herbal medicine. *Data collection and analysis:* Three authors independently assessed risk of bias and extracted data; we contacted study authors for additional information. Meta-analyses were not performed as only one study was included. The primary outcome measure was decrease in pain from endometriosis. Secondary outcome measures included improvement in quality of life scores, pregnancy rate, adverse effects and rate of endometriosis recurrence.

**MAIN RESULTS:**

Twenty-four studies were identified that involved acupuncture for endometriosis; however only one trial, enrolling 67 participants, met all the inclusion criteria. The single included trial defined pain scores and cure rates according to the Guideline for Clinical Research on New Chinese Medicine. Dysmenorrhoea scores were lower in the acupuncture group (mean difference -4.81 points, 95% confidence interval -6.25 to -3.37, P < 0.00001) using the 15-point Guideline for Clinical Research on New Chinese Medicine for Treatment of Pelvic Endometriosis scale. The total effective rate ('cured', 'significantly effective' or 'effective') for auricular acupuncture and Chinese herbal medicine was 91.9% and 60%, respectively (risk ratio 3.04, 95% confidence interval 1.65 to 5.62, P = 0.0004). The improvement rate did not differ significantly between auricular acupuncture and Chinese herbal medicine for cases of mild to moderate dysmenorrhoea, whereas auricular acupuncture did significantly reduce pain in cases of severe dysmenorrhoea. Data were not available for secondary outcomes measures.

**AUTHORS' CONCLUSIONS:**

The evidence to support the effectiveness of acupuncture for pain in endometriosis is limited, based on the results of only a single study that was included in this review. This review highlights the necessity for developing future studies that are well-designed, double-blinded, randomized controlled trials that assess various types of acupuncture in comparison to conventional therapies.

**This is the abstract of a Cochrane Review published in the Cochrane Database of Systematic Reviews (CDSR) 2011, issue 9, Art. No. CD007864. DOI:** 10.1002/14651858.CD007864.pub2 (http://cochrane.bvsalud.org/cochrane/main.php?lib=COC&searchExp=Acupuncture%20and%20for%20and%20pain%20and%20in%20and%20endometriosis&lang=pt).

**The full text is available from:** http://onlinelibrary.wiley.com/doi/10.1002/14651858.CD007864.pub2/abstract.

**The abstract is also available in the Portuguese, French and Spanish languages from:** http://summaries.cochrane.org/pt/CD007864/acupuntura-para-dor-da-endometriose.
